# Molecular epidemiology of serotype 19A *Streptococcus pneumoniae* among invasive isolates from Alaska, 1986–2010

**DOI:** 10.3402/ijch.v72i0.20854

**Published:** 2013-08-05

**Authors:** Karen Rudolph, M.G. Bruce, L. Bulkow, T. Zulz, A. Reasonover, M. Harker-Jones, D. Hurlburt, T.W. Hennessy

**Affiliations:** Arctic Investigations Program, Division of Preparedness and Emerging Infections, National Center for Emerging and Zoonotic Infectious Diseases, Centers for Disease Control and Prevention (CDC)

**Keywords:** Streptococcus pneumoniae, serotype 19A, MLST, invasive disease, Alaska

## Abstract

**Background:**

After the introduction of the 7-valent pneumococcal conjugate vaccine (PCV7) in Alaska, the incidence of invasive pneumococcal disease (IPD) due to non-vaccine serotypes, particularly serotype 19A, increased. The aim of this study was to describe the molecular epidemiology of IPD due to serotype 19A in Alaska.

**Methods:**

IPD data were collected from 1986 to 2010 through population-based laboratory surveillance. Isolates were serotyped by the Quellung reaction and MICs determined by broth microdilution. Genotypes were assessed by multilocus sequence typing.

**Results:**

Among 3,294 cases of laboratory-confirmed IPD, 2,926 (89%) isolates were available for serotyping, of which 233 (8%) were serotype 19A. Across all ages, the proportion of IPD caused by serotype 19A increased from 3.5% (63/1823) pre-PCV7 (1986–2000) to 15.4% (170/1103) post-PCV7 (2001–2010) (p<0.001); among children <5 years of age, the proportion increased from 5.0% (39/776) to 33.0% (76/230) (p<0.001). The annual incidence rate of IPD due to serotype 19A (all ages) increased from 0.73 cases pre-PCV7 to 2.56 cases/100,000 persons post-PCV7 (p<0.001); rates among children <5 years of age increased from 4.84 cases to 14.1 cases/100,000 persons (p<0.001). Among all IPD isolates with reduced susceptibility to penicillin, 17.8% (32/180) were serotype 19A pre-PCV7 and 64% (121/189) were serotype 19A post-PCV7 (p<0.001). Eighteen different sequence types (STs) were identified; ST199 or single locus variants of ST199 (n=150) and ST172 (n=59) accounted for the majority of isolates. Multidrug-resistant isolates were clustered in ST199 and ST320.

**Conclusion:**

While PCV13 should significantly reduce the burden of disease due to 19A, these data highlight the need to continue surveillance for IPD to monitor the effects of vaccination on the expansion and emergence of non-PCV strains.

*Streptococcus pneumoniae* is a major cause of invasive diseases, such as bacteremia, septicemia and meningitis, and less severe conditions such as middle-ear infections, sinusitis and recurrent bronchitis. Worldwide, pneumococci cause over 1 million deaths annually, mainly among children <5 years of age, mostly in developing countries. In addition to being a major cause of morbidity and mortality worldwide, pneumococci are also becoming more resistant to commonly used antibiotics. One of the strategies used to reduce both the incidence of invasive disease and the spread of antibiotic-resistant isolates is administration of vaccines directed against the polysaccharide capsule. In children <2 years of age, where the greatest burden of disease exists, recent efforts to develop effective pneumococcal vaccines have been concentrated on conjugate vaccines in which the capsular polysaccharides are covalently coupled to carrier proteins. The first such conjugate vaccine (PCV7), which was introduced into the universal immunization program in the United States in 2000, contained the 7 most common pneumococcal serotypes (4, 6B, 9V, 14, 18C, 19F and 23F) causing invasive disease in infants and young children prior to its introduction in 2000. After the introduction of the 7-valent pneumococcal conjugate vaccine (PCV7), the incidence of invasive pneumococcal disease (IPD) caused by vaccine serotypes decreased significantly among both children and adults (1–3). However, rates of IPD caused by non-vaccine serotypes, particularly serotype 19A, increased (4–10).

Prior to the introduction of PCV7 into the routine vaccination schedule in Alaska, rates of invasive pneumococcal disease among Alaska Native persons were among the highest reported, particularly among Native children <2 years of age where rates were 3.5 times higher than observed in non-Native children of the same age (11, 12). Following the introduction of PCV7 in Alaska in 2001, the rate of vaccine-type invasive disease among Alaska Native children <2 years of age declined by 92% with a similar decline in rates among non-Native children of the same age (9). In addition, invasive disease due to antibiotic-resistant serotypes decreased, however, our statewide surveillance data from 2001 to 2010 revealed an increase in IPD due to serotype 19A among all age groups. Here we describe the antimicrobial susceptibilities and genetic backgrounds of serotype 19A IPD isolates in the 15 years before the introduction of PCV7 and in the 10 years following vaccine introduction.

## Material and methods

### Population studied

Alaska's population of 710,000 (2010 US census) includes 142,000 (20%) Alaska Native (AN) and American Indian peoples, with 7,100 younger than 2 years of age. Sixty-five percent of Alaska Native peoples live in rural communities, many of which are isolated villages with populations ranging from 50 to 1000 persons. Health care for the AN population is provided through a statewide tribally-operated health delivery system which includes community health practitioners at the village level, primary care providers in regional hub communities and a referral hospital in Anchorage.

### Invasive disease surveillance (1986–2010)

The Arctic Investigations Program (AIP) established a population-based statewide surveillance system to monitor invasive pneumococcal disease (IPD) in 1986. Isolates of *S. pneumoniae* are received at the AIP laboratory, Anchorage, AK, from 23 regional hospital laboratories processing sterile site specimens (blood and cerebrospinal, pleural, peritoneal or joint fluid) in the state. Pneumococci were confirmed by colony morphology, susceptibility to optochin (Difco, Detroit) and bile solubility. All isolates were serotyped by slide agglutination and confirmed by the Quellung reaction using group- and type-specific antisera (Staten Serum Institute, Copenhagen, Denmark).

### Antimicrobial susceptibility testing

Susceptibility testing was performed by use of the broth microdilution method as described by the Clinical and Laboratory Standards Institute (CLSI) for penicillin, erythromycin, trimethoprim-sulfamethoxazole (TS), tetracycline, chloramphenicol, ceftriaxone, cefotaxime, vancomycin and rifampin (13). The MIC was determined to be the lowest concentration of antibiotic that inhibited growth. The 2007 interpretive CLSI guidelines were used to define non-susceptibility and included isolates that were either intermediately resistant or fully resistant to a particular antibiotic (13). Multidrug-resistant isolates were defined as having intermediate or full resistance to three or more different classes of antibiotics.

### Macrolide resistance genotypes

Erythromycin-resistant serotype 19A isolates were analysed for the presence of *erm*B and *mef* genes using a duplex PCR assay. Bacterial cells were suspended in 0.1 ml of nuclease free H_2_O, heated at 100°C for 10 min, centrifuged at 13,000 rpm for 5 min and stored at −30°C until used. Erythromycin-resistance genes *erm*B and *mef* were screened using primer sets designed by Sutcliff et al. and Tait-Kamradt et al., respectively (14, 15). PCR was performed in a total volume of 25 µl consisting of 12.5 µl AmpliTaq Gold^®^ (Applied Biosystems, Foster City, CA), 3.0 µl of DNA lysate, 0.5 µM of each *erm*B primer and 2.0 µM of each *mef* primer. Amplifications were performed in a Perkin-Elmer GeneAmp PCR system 9700 (Applied Biosystems) under the following conditions: 94°C for 3 min followed by 30 cycles of 94°C for 45 s, 53°C for 30 s and 72°C for 2 min with a final extension at 72°C for 5 min. Negative controls contained the PCR mixture without the DNA template; positive controls contained *S. pneumoniae* DNA from erythromycin-resistant strains known to carry either *mef* or *erm*B. Amplification products were run through 3% ReadyAgarose™ gels (Bio-Rad, Hercules, CA) in 1X TAE (Invitrogen, Carlsbad, CA) at 100 V for 60 min. PCR product size for the *erm*B and *mef* genes are 639 bp and 940 bp, respectively. The PCR products of the *mef* gene were digested with *Dra*I (Promega, Madison, WI) in order to discriminate between the *mef*A and *mef*E subclasses (16).

### MLST analysis

Multilocus sequence typing (MLST) was performed as previously described (17), with modifications (7) on all viable serotype 19A isolates recovered from 1986–2010. The sequence types (ST) were determined by comparing the sequences with alleles downloaded from the pneumococcal MLST database (http://spneumoniae.mlst.net). Clonal complexes were assigned using the eBURST algorithm with the software available at the MLST website (http://www.mlst.net).

### Statistical analysis

Disease rates by period were compared using mid-P exact significance. Proportions of isolates were compared using Chi-square or Fisher's exact test as appropriate. Analyses were performed with Stata Version 10. All p-values are two-sided and a p-value of <0.05 is considered statistically significant.

## Results

### Bacterial isolates and incidence

From 1986 to 2010, 3294 cases of culture-confirmed invasive pneumococcal disease (IPD) were reported to AIP. Of these cases, 2926 (89%) isolates were available for serotyping of which 233 (8%) were serotype 19A. Rates of IPD due to serotype 19A increased significantly (p<0.001) after the introduction of PCV7 in Alaska. The annual incidence rate of IPD due to serotype 19A among all ages increased from 0.73 cases per 100,000 persons pre-PCV7 (1986–2000) to 2.56 cases per 100,000 persons post-PCV7 (2001–2010) (p<0.001) (Table I). The annual incidence rate of IPD due to serotype 19A among children <5 years of age increased from 4.84 cases per 100,000 persons pre-PCV7 to 14.1 cases per 100,000 persons post-PCV7 (p<0.001). The proportion of IPD caused by serotype 19A among all age groups increased from 3% (63/1823) pre-PCV7 to 15% (170/1103) post-PCV7 (p<0.0001). Among children <5 years of age, the proportion of IPD caused by serotype 19A increased from 5% (39/776) pre-PCV7 to 33% (76/230) post-PCV7 (p<0.0001). Common clinical presentations among serotype 19A IPD cases included pneumonia with bacteremia (156, 67%), bacteremia (57, 24%) and meningitis (12, 5%).

**Table I:** Annual incidence of serotype 19A and non-serotype 19A IPD in Alaska, pre-PCV7 (1986–2000) and post-PCV7 (2001–2010)

	Number of cases (rate per 100,000 population)					
			
	1986–2000		2001–2010		p-Value for change in rate	
			
Age (yrs)	19A	Non-19A serotypes	19A	Non-19A serotypes	19A	Non-19A serotypes
<5	39 (4.84)	737 (91.5)	76 (14.1)	154 (28.5)	**<0.001**	**<0.001**
5+	24 (0.31)	1023 (13.1)	94 (1.54)	779 (12.7)	**<0.001**	0.611
Total	63 (0.73)	1760 (20.4)	170 (2.56)	933 (14.0)	**<0.001**	**<0.001**

A p-value of <0.05 is considered statistically significant.

### MLST

**Fig. 1:**
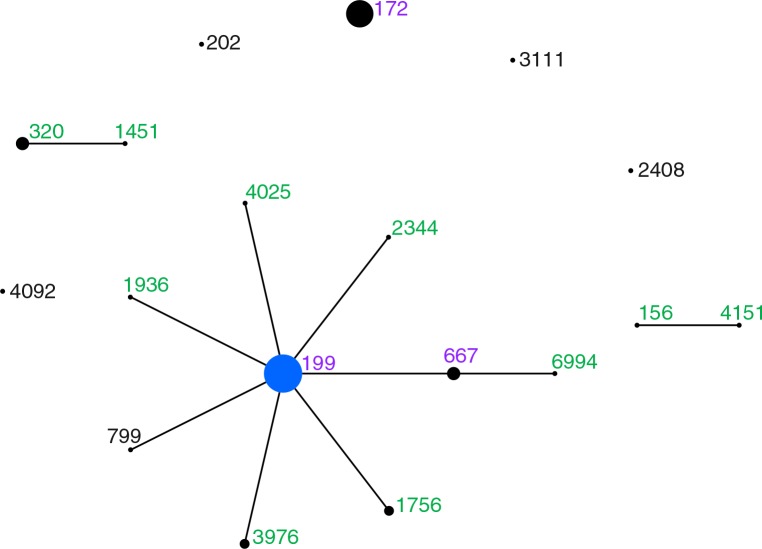
Population snapshot of serotype 19A isolates in Alaska comparing sequence types (ST) found in 1986–2000 and 2001–2010. Each circle represents a single ST, with the area proportional to the number of isolates of that type. Solid lines between STs represent single-locus variants. STs in black are STs found only in the pre-PCV7 (1986–2000) era. STs in green are STs found only in the post-PCV7 (2001–2010) era. STs in pink are STs found in both the pre-PCV7 and post-PCV7 eras.

Of the 233 serotype 19A isolates, 231 were available for molecular typing. MLST analysis revealed 18 sequence types (ST) (Fig. 1). Six STs were unique to the 1986–2000 isolates and nine STs were unique to the 2001–2010 isolates. Four STs (3976, 4092, 4151, 6994) were new to the MLST database at the time of this analysis. Of the nine STs found among the serotype 19A isolates recovered during the pre-PCV7 period (1986–2000), three STs (199, 667, 172) accounted for 90% (55/61) of the isolates. Of the 12 STs found among the serotype 19A isolates recovered during the post-PCV7 period (2001–2010), only three STs (199, 667, 172) were found in the pre-PCV7 period. These three STs accounted for 76% (130/170) of the serotype 19A isolates recovered in this time period. Among the remaining 40 serotype 19A isolates recovered during the post-PCV7 period, three STs (320, 1756, 3976) accounted for 85% (33/40) of the isolates.

The most frequent ST across the entire time period was ST199 and, along with closely related single- and double-locus variants form clonal complex (CC) 199, accounting for the majority (65%, 150/231) of the isolates (Fig. 1). However, the proportion of CC199 isolates decreased from 90% (55/61) in 1986–2000 to 56% (95/170) in 2001–2010, which was statistically significant (p<0.001) (Table II). The second most frequent ST was ST172 which accounted for 26% (59/231) of the isolates. ST172 isolates were first seen in 2000 and increased significantly over time. By the later part of the post-PCV7 era (2006–2010), ST172 isolates accounted for 33% of serotype 19A isolates. During this same time period, we observed the introduction of CC320 (ST320 and ST1451) isolates into this population. These isolates accounted for 13% (15/118) of the serotype 19A isolates in this time period.

**Table II:** Distribution of serotype 19A genotypes in Alaska by time period, 1986–2010

	Number of isolates (% in time period)				
	
CC or ST^a^	1986–1994	1995–2000	2001–2005	2006–2010	p-value
199	38 (95%)	17 (81%)	33 (63%)	62 (53%)	<0.001
172	0	2 (9.5%)	18 (35%)	39 (33%)	<0.001
320	0	0	0	15 (13%)	0.001
156	0	0	1 (1.9%)	2 (1.7%)	
202	0	1 (4.8%)	0	0	
3111	1 (2.5%)	0	0	0	
2408	1 (2.5%)	0	0	0	
4092	0	1 (4.8%)	0	0	
Total	40	21	52	118	

a CC/ST=clonal complex or sequence type.

A total of 40% of CC320 isolates were recovered from children <2 years of age and, while this proportion was higher than for ST172 and CC199 isolates, it was not statistically significant. Likewise, we did not observe statistically significant differences with regards to clinical syndromes, sex, or case fatality when comparing IPD caused by CC320 isolates with IPD caused by the other serotype 19A genotypes.

### Antimicrobial susceptibility

Among all IPD isolates with reduced susceptibility to penicillin, 18% (32/180) were serotype 19A pre-PCV7 and 64% (121/189) were serotype 19A post-PCV7 (p<0.001). The proportion of serotype 19A isolates that were non-susceptible to penicillin (MIC≥0.12 µg/ml), erythromycin, and TS increased significantly in the post-PCV7 era (Table III). While not statistically significant, we also observed an increase in the proportion of serotype 19A isolates with reduced susceptibility to cefotaxime and ceftriaxone. Among all IPD isolates that were multidrug resistant, 4% (4/109) were serotype 19A pre-PCV7 and 49% (37/76) were serotype 19A post-PCV7. Among the 45 serotype 19A isolates that were non-susceptible to erythromycin, 28 (62%) carried the *mef*E gene. Both the *mef*E and *erm*B genes were carried by all of the CC320 serotype 19A isolates.

**Table III:** Proportion of IPD isolates non-susceptible to antibiotics by time period, 1986–2000 vs. 2001–2010, Alaska

	Serotype 19A			All other serotypes		
	Number of isolates (%)			Number of isolates (%)		
		
	1986–2000	2001–2010		1986–2000	2001–2010	
Antibiotic	N=63	N=170	p-value	N=1760	N=897	p-value
Penicillin	32 (52%)	121 (71%)	0.005	148 (8%)	68 (8%)	0.460
Tetracycline	2 (3%)	16 (9%)	0.166	49 (3%)	28 (3%)	0.617
Erythromycin	3 (5%)	45 (27%)	<0.001	153 (9%)	68 (8%)	0.328
TS^a^	12 (22%)	126 (74%)	<0.001	283 (16%)	109 (12%)	0.007
Cefotaxime	3 (5%)	13 (7%)	0.327	59 (3%)	22 (3%)	0.204
Ceftriaxone	3 (5%)	14 (9%)	0.411	39 (2%)	14 (2%)	0.607
3 or more classes	4 (6%)	35 (21%)	0.010	105 (6%)	39 (4%)	0.083

a TS=trimethoprim-sulfamethoxazole.

## Discussion

Prior to the introduction of PCV7, rates of IPD among Alaska Native children were among the highest reported in the world with vaccine serotypes accounting for 74% of known serotype IPD (11, 12, 18). PCV7 was introduced into the routine childhood vaccine schedule for all Alaskan children on January 1, 2001. Within three years, rates of vaccine-type IPD had decreased by 95% and total IPD rates had declined by 65% among Alaska Native children <2 years of age (18). While the overall rate of IPD decreased following the introduction of PCV7, rates of IPD caused by non-vaccine serotypes increased 140% compared with the prevaccine period (9). This increase was in large part due to an increase in IPD caused by serotype 19A. When compared to the pre-PCV7 era, the proportion of IPD caused by serotype 19A increased 5-fold from 3.5 to 15.4% across all age groups. Among children <5 years of age, the group targeted for vaccination with PCV7, the proportion increased nearly 7-fold from 5 to 33%. Since the introduction of PCV7, there have been numerous reports describing increased rates of IPD with non-vaccine serotypes, particularly serotype 19A (4–8, 19–25). From these reports, a number of speculations around possible mechanisms for the increased incidence of IPD caused by serotype 19A have emerged and include capsular switching of vaccine serotypes under selective pressure by PCV7, expansion of highly resistant strains under antibiotic pressure, introduction of new clones and secular changes in serotype frequencies to name but a few. It should be noted that the increased incidence of serotype 19A IPD in various populations is most likely due to a combination of multiple factors rather than a single factor.

Examination of the genetic structure of our 19A isolates revealed that the increase in serotype 19A IPD was the result of the expansion of pre-existing STs (ST199 and closely related variants and ST172) and the emergence of serotype 19A STs associated with multidrug resistance, in particular, ST320. However, despite changes in the 19A population structure in the post-PCV7 era, CC199 isolates remained the most common genotype. Prior to the introduction of PCV7, 90% of our 19A isolates were CC199 isolates. This finding is similar to what was reported by Beall et al. who found that >70% of serotype 19A isolates recovered in 1999 from the Centers for Disease Control and Prevention's Active Bacterial Core (ABC) surveillance sites were CC199 (19). Following the introduction of PCV7, while CC199 isolates remained predominant among serotype 19A isolates recovered from children <5 years of age in 2003–2004, by 2005 CC199 isolates had declined to 59% of 19A isolates. In Alaska, CC199 serotype 19A isolates also began declining, dropping to 63% in 2001–2005 and 53% in 2006–2010. CC199 has been the predominant CC in several other studies both prior to and after the introduction of PCV7 (6, 8, 23, 24, 26, 27).

In contrast to CC199 serotype 19A isolates, isolates of ST172 which were first seen in Alaska in 2000, emerged rapidly after introduction of PCV7 and accounted for 34% of 19A isolates from 2001 to 2010. ST172 isolates have also been reported among serotype 19A isolates from the ABC's surveillance both in the pre- and post-PCV7 eras but accounted for a much lower proportion of isolates (7, 19, 28). In contrast, ST172 isolates constituted 69.4% of all tested serotype 19A acute otitis media (AOM) isolates recovered from Jewish children during an 8-year study period (1999–2006) (29). In a more recent study, ST172 was also found to be predominant among serotype 19A IPD isolates from this same population (30). ST172 isolates within the pneumococcal MLST database have also been associated with serotypes 6A, 6B, 6C, 15B, 19F, 23B, 23F and 35B. This suggests that capsular switching may occur more frequently than expected as a number of different serotypes share the same ST.

The post-PCV7 increase in serotype 19A IPD in Alaska was also driven by the emergence of ST320 isolates late in the PCV7 era. ST320 is a double-locus variant of the globally dispersed Taiwan^19F^-14 ST236 clone, which is now part of CC320/271 and also contains ST236 (7). In an ABC's survey conducted in 2003–2004, investigators found that CC320/271 accounted for 13.4% of the serotype 19A IPD cases in the US (5). The prevalence of CC320/271 isolates in the US continued to increase and by 2007 had reached 33%. In Alaska, CC320/271 (ST320 and ST 1451) isolates were not seen until 2007 but by 2010, made up 28% of the serotype 19A isolates. The emergence of CC320/271 among serotype 19A isolates in the post-PCV7 era has not been restricted to the US and in fact occurred simultaneously in many different countries (20, 22, 23, 31, 32). In contrast, South Korea reported finding ST320 among both serotype 19F and 19A isolates in the 1990s, well before PCV7 was introduced in that population in 2003 (33). It remains unclear as to why ST320 serotype 19A isolates would emerge without vaccine pressure against serotype 19 but does suggest that vaccination may not be the only factor responsible for the observed increase in serotype 19A. Other populations have also seen an increase in the incidence of serotype 19A in the absence of vaccine pressure which provides further evidence that a number of factors, including antibiotic use, may be involved in the emergence of non-vaccine types (29).

Prior to the introduction of PCV7 in Alaska, PCV7 serotypes accounted for 86% of invasive isolates non-susceptible to penicillin, erythromycin, or TMP-SMX (12). Consequently, after PCV7 introduction the proportion of isolates with reduced susceptibility to these antimicrobials declined (9). Similarly, among ABC surveillance sites, rates of IPD caused by penicillin-non-susceptible strains and strains non-susceptible to multiple antibiotics decreased between 1999 and 2004 among both children and adults (35). However, during this same time period, the proportion of penicillin-non-susceptible serotype 19A isolates increased significantly (7). In addition, the proportion of serotype 19A isolates that were non-susceptible to other antibiotics and multiple antibiotics increased and was seen in the US and elsewhere (7, 20, 23, 29, 30). Similar trends were seen in Alaska where the proportion of serotype 19A isolates with reduced susceptibility to penicillin increased from 52% in the pre-PCV7 era to 71% in the post-PCV7 era. We also observed a significant increase in the proportion of serotype 19A isolates that were resistant to erythromycin, TS and multiple antibiotics.

The increase in antibiotic non-susceptibility among the serotype 19A isolates that we observed in the post-PCV7 era was the result of a shift in the genetic structure of the 19A population. The increase in penicillin non-susceptibility was the result of an increase in ST172, which was typically intermediately resistant to penicillin and CC320 isolates which were fully resistant to penicillin (MIC≥2 µg/ml). In addition to penicillin resistance, CC320 isolates were resistant to tetracycline, cefotaxime, ceftriaxone, erythromycin and TMP-SMX. Multidrug resistance among CC320/271 isolates has been reported by others and suggests that this high-level antimicrobial resistance phenotype may have played a role in the emergence of this clone (6, 20, 23, 28, 32). The increase in erythromycin and TS-non-susceptibility was also the result of an increase in non-susceptibility among CC199 isolates. Prior to the introduction of PCV7, CC199 were typically susceptible to all antibiotics or non-susceptible to penicillin and/or TS. In the late PCV7-era, erythromycin-resistant CC199 isolates were recovered. All of these isolates carried the *mef*E gene which was, in contrast to the CC320 isolates which carried both *mef*E and *erm*B genes, a phenotype commonly seen among the Taiwan^19F^-14 ST236 clone (33).

In conclusion, the increase in serotype 19A IPD in Alaska was driven by the expansion of clones existing prior to PCV7 introduction, particularly ST172 and the emergence of multidrug-resistant clones that evolved as a result of capsule switching. Serotype 19A is included in the recently licensed PCV13 vaccine, and as expected, we have seen a decline in IPD caused by 19A in Alaska (34). However, since other serotypes with similar characteristics and disease potential may be the next in line to expand, surveillance of serotypes, sequences types and antimicrobial resistance remain important activities to continue as we introduce higher valency pneumococcal conjugate vaccines.

## References

[1] Whitney CG, Farley MM, Hadler J, Harrison LH, Bennett NM, Lynfield R (2003). Decline in invasive pneumococcal disease after the introduction of protein-polysaccharide conjugate vaccine. N Engl J Med.

[2] Kaplan SL, Mason EO, Wald ER, Schutze GE, Bradley JS, Tan TQ (2004). Decrease of invasive pneumococcal infections in children among 8 children's hospitals in the United States after the introduction of the 7-valent pneumococcal conjugate vaccine. Pediatrics.

[3] Lexau CA, Lynfield R, Danila R, Pilishvili T, Facklam R, Farley MM (2005). Changing epidemiology of invasive pneumococcal disease among older adults in the era of pediatric pneumococcal conjugate vaccine. JAMA.

[4] Hicks L, Harrison L, Flannery B, Hadler JL, Schaffner W, Craig AS (2007). Incidence of pneumococcal disease due to non-pneumococcal conjugate vaccine (PCV7) serotypes in the United States during the era of widespread PCV7 vaccination, 1998–2004. J Infect Dis.

[5] Pai R, Moore MR, Pilishvili T, Gertz RE, Whitney CG, Beall B (2005). Post-vaccine genetic structure of *Streptococcus pneumoniae* serotype 19A from children in the United States. J Infect Dis.

[6] Pelton SI, Huot H, Finkelstein JA, Bishop CJ, Hsu KK, Kellenbery J (2007). Emergence of 19A as virulent and multidrug resistant pneumococcus in Massachusetts following universal immunization of infants with pneumococcal conjugate vaccine. Pediatr Infect Dis J.

[7] Moore MR, Gertz RE, Woodbury RL, Barkocy-Gallagher GA, Schaffner W, Lexau C (2008). Population snapshot of emergent *Streptococcus pneumoniae* serotype 19A in the United States, 2005. J Infect Dis.

[8] Messina AF, Katz-Gaynor K, Barton T, Ahmad N, Ghaffar F, Rasko D (2007). Impact of the pneumococcal conjugate vaccine on serotype distribution and antimicrobial resistance of invasive *Streptococcus pneumoniae* isolates in Dallas, TX, children from 1999 through 2005. Pediatr Infect Dis J.

[9] Singleton RJ, Hennessy TW, Bulkow LR, Hammitt LL, Zulz T, Hurlburt DA (2007). Invasive pneumococcal disease caused by non-vaccine serotype among Alaska Native children with high levels of 7-valent pneumococcal conjugate vaccine coverage. JAMA.

[10] Centers for Disease Control and Prevention (2007). Emergence of antimicrobial-resistant serotype 19A *Streptococcus pneumoniae* – Massachusetts, 2001–2006. MMWR Morb Mortal Wkly Rep.

[11] Davidson M, Parkinson AJ, Bulkow LR, Fitzgerald MA, Peters HV, Parks DJ (1994). The epidemiology of invasive pneumococcal disease in Alaska, 1986–1990 – ethnic differences and opportunities for prevention. J Infect Dis.

[12] Rudolph KM, Parkinson AJ, Reasonover AL, Bulkow LR, Parks DJ, Butler JC (2000). Serotype distribution and antimicrobial resistance patterns of invasive isolates of *Streptococcus pneumoniae*: Alaska, 1991–1998. J Infect Dis.

[13] (2007). Clinical and Laboratory Standards Institute. Performance standards for antimicrobial susceptibility testing: 15th Informational Supplement.

[14] Sutcliffe J, Grebe T, Tait-Kamradt A, Wondrack L (1996). Detection of erythromycin resistant determinants by PCR. Antimicrob Agents Chemother.

[15] Tait-Kamradt A, Clancy J, Cronan M, Dib-Hajj F, Wondrack L, Yuan W (1997). *mef*E is necessary for the erythromycin-resistant M phenotype in *Streptococcus pneumoniae*. Antimicrob Agents Chemother.

[16] Montanari MP, Mingoia M, Cochetti I, Varaldo PE (2003). Phenotypes and genotypes of erythromycin-resistant pneumococci in Italy. J Clin Micro.

[17] Enright MC, Spratt BG (1998). A multilocus sequence typing scheme for *Streptococcus pneumoniae*: identification of clones associated with serious invasive disease. Microbiology.

[18] Hennessy TW, Singleton RJ, Bulkow LR, Bruden DL, Hurlburt DA, Parks D (2005). Impact of heptavalent pneumococcal conjugate vaccine on invasive disease, antimicrobial resistance and colonization in Alaska Natives: progress towards elimination of a health disparity. Vaccine.

[19] Beall B, McEllistrem MC, Gertz RE, Wedel S, Boxrud DJ, Gonzalez AL (2006). Pre- and postvaccination clonal compositions of invasive pneumococcal serotypes of isolates collected in the United States in 1999, 2001, and 2002. J Clin Micro.

[20] Pillai DR, Shahinas D, Buzina A, Pollock RA, Lau R, Khairnar K (2009). Genome-wide dissection of globally emergent multi-drug resistant serotype 19A *Streptococcus pneumoniae*. BMC Genomics.

[21] Hanage WP, Bishop CJ, Lee GM, Lipsitch M, Stevenson A, Rifas-Shiman SL (2011). Clonal replacement among 19A *Streptococcus pneumoniae* in Massachusetts, prior to 13 valent conjugate vaccination. Vaccine.

[22] Mahjoub-Messai F, Doit C, Koeck J, Billard T, Evrard B, Bidet P (2009). Population snapshot of *Streptococcus pneumoniae* serotype 19A isolates before and after introduction of seven-valent pneumococcal vaccination for French children. J Clin Microbiol.

[23] Muñoz-Almagro C, Esteva C, de Sevilla M, Selva L, Gene A, Pallares R (2009). Emergence of invasive pneumococcal disease caused by multidrug-resistant serotype 19A among children in Barcelona. J Infect.

[24] Vestrheim DF, Steinbakk M, Aaberge IS, Caugant DA (2012). Postvaccination increase in serotype 19A pneumococcal disease in Norway is driven by expansion of penicillin-susceptible strains of the ST199 complex. Clin Vaccine Immunol.

[25] van Deursen AMM, van Mens SP, Sanders EAM, Vlaminckx BJM, de Melker HE, Schouls LM (2012). Invasive pneumococcal disease and 7-valent pneumococcal conjugate vaccine, the Netherlands. Emerg Infect Dis.

[26] Clarke SC, Scott KJ, McChlery SM (2004). Serotypes and sequence types of pneumococci causing invasive disease in Scotland prior to the introduction of pneumococcal conjugate polysaccharide vaccines. J Clin Micro.

[27] Scott JR, Hanage WP, Lipsitch M, Millar EV, Moulton LH, Hinds J (2012). Pneumococcal sequence type replacement among American Indian children: a comparison of pre- and routine-PCV7 eras. Vaccine.

[28] Beall BW, Gertz RE, Hulkower RL, Whitney CG, Moore MR, Brueggemann AB (2011). Shifting genetic structure of invasive serotype 19A pneumococci in the United States. J Infect Dis.

[29] Dagan R, Givon-Lavi N, Leibovitz E, Greenberg D, Porat N (2009). Introduction and proliferation of multidrug-resistant *Streptococcus pneumoniae* serotype 19 A clones that cause acute otitis media in an unvaccinated population. J Infect Dis.

[30] Porat N, Benisty R, Trefler R, Givon-Lavi N, Dagan R (2012). Clonal distribution of common pneumococcal serotypes not included in the 7-valent conjugate vaccine (PCV7): marked differences between two ethnic populations in southern Israel. J Clin Microbiol.

[31] Tarragó D, Aguilar L, García R, Gimenez M, Granizo J, Fenoll A (2011). Evolution of clonal and susceptibility profiles of serotype 19A *Streptococcus pneumoniae* among invasive isolates from children in Spain, 1990 to 2008. Antimicrob Agents Chemo.

[32] Ardanuy C, Rolo D, Fenoll A, Tarrago D, Calatayud L, Liñares J (2009). Emergence of a multidrug resistant clone (ST320) among invasive serotype 19A pneumococci in Spain. J Antimicrob Chemother.

[33] Choi EH, Kim SH, Eun BW, Kim SJ, Kim NH, Lee J (2008). *Streptococcus pneumoniae* serotype 19A in children, South Korea. Emerg Infect Dis.

[34] Bruce M, Wenger J, Zulz T, Singleton R, Bulkow L, Rudolph K (2012). Invasive pneumococcal disease (IPD) in Alaska children: impact of the 13-valent pneumococcal conjugate vaccine (PCV13). Presented at 8th International Symposium on Pneumococci and Pneumococcal Diseases (ISPPD), Iguacu Falls, Brazil.

[35] Kyaw MH, Lynfield R, Schaffner W, Craig AS, Hadler J, Reingold A (2006). Effect of introduction of the pneumococcal conjugate vaccine on drug-resistant *Streptococcus pneumoniae*. N Engl J Med.

